# 

**Published:** 1886-07

**Authors:** 


### BOOKS AND PAMPHLETS RECEIVED.

STUDIES FROM THE BIOLOGICAL LABORATORY, JOHNS HOPKINS UNIVERSITY, Baltimore. Edited by H. Newell Martin, M. D., F. R. S., and W. K. Brooks, Ph.D. Vol. III., Nos. 5, 6 and 7. 1886.

JOHNS HOPKIN'S UNIVERSITY CIRCULARS. Baltimore, 1886.

ANNUAL ANNOUNCEMENT of the New York College of Veterinary Surgeons, and School of Comparative Medicine. Session 1886-7.

CATALOGUE AND ANNOUNCEMENT University of Pennsylvania, 1885-86. Department of Biology, and Auxiliary Department of Medicine.

CATALOGUE Meharry Medical Department of Tennessee College, 11th Annual Announcement. Nashville, 1885-86.

CORNELL UNIVERSITY REGISTER, 1885-86. Ithaca.

ON THE LIMITATION OF THE CONTAGIOUS STATE OF SYPHILIS, ESPECIALLY, IN ITS RELATION TO MARRIAGE, G. F. N. Otis, M. D. New York: Wm. Wood & Co., 1886.

FIRST ANNUAL REPORT of the Bureau of Animal Industry, for the year 1884, Washington, 1885.

THIRD ANNUAL ANNOUNCEMENT of the School of Pharmacy, Purdue University, 1886-87. Lafayette, 1886.

PURDUE UNIVERSITY, School of Pharmacy, Bulletin No. 1.

BULLETIN of the Brookville Society of Natural History, No. 2. Richmond, Ind., 1886.

A MANUAL of the Diseases of the Elephant and of his Management and Uses, by John Henry Steel, A D.V., M.R.C.V.S., Co-editor of the Quarterly Journal of Veterinary Science in India; illustrated. Madras, 1885. Review in October number of Journal.

ANNOUNCEMENT of the New York Society for the Prevention of Contagious Diseases. June, 1886.

HATTER'S CONSUMPTION, by J. W. Stickler, M.S., M.D. Reprint from the New York Medical Journal.

